# Influence of Fluid Food Viscosity on Internal Flow Characteristics of Conveying Pump

**DOI:** 10.3389/fnut.2022.910589

**Published:** 2022-06-09

**Authors:** XiaoQi Jia, Qingyang Chu, ZuChao Zhu, Qiangmin Ding, Panlong Gao

**Affiliations:** ^1^Key Laboratory of Fluid Transmission Technology of Zhejiang Province, Zhejiang Sci-Tech University, Hangzhou, China; ^2^Hefei General Machinery Research Institute, Hefei, China

**Keywords:** external characteristic, pressure pulsation, radial force, entropy production, viscosity, conveying pump

## Abstract

A fluid food conveying pump is used to convey edible or nutritional fluids and semi-fluids (containing suspended soft and hard particles and with different viscosities), such as water, glycerin, yogurt, and juice concentrate. Since different fluid food have different viscosities, the internal flow characteristics and conveying performance of food conveying pump are greatly affected by viscosity. To obtain the influence law of fluid food viscosity on the internal flow characteristics of the pump, the internal flow characteristics of food conveying pump when conveying food of 4 different viscosities (water, glycerin, 67.2 °Bx wild jujube juice, and 71.0 °Bx haw juice) were compared and observed in this study. The results showed that, with the increase in food viscosity, the overall flow loss in the pump, the entropy generation, and the proportion of total entropy generation in the pump chamber increase, but the conveying performance of the food conveying pump gets worse; however, the pressure pulsation intensity caused by static and dynamic interferences decreases with the increase in viscosity.

## Introduction

The processing and manufacturing of food is a critical part of the food industry. Making raw materials into semi-finished and finished products requires complicated processes. A pump is an indispensable part in the manufacturing of fluid food. It pressurizes fluid food and conveys it to each production link. Since the internal flow characteristics and conveying performance of a conveying pump are greatly affected by the fluid food viscosity, in order to ensure smooth food processing, it is very important to study the influence of viscosity on the internal flow and conveying characteristics of the pump during food conveying. The main flow losses in the food conveying pump are caused by viscous dissipative vortex flow, mainly including backflow (1), jet flow-wake flow (2), secondary flow (3), and shedding vortex (4). In Li (5), the stable-state flow of fluids of different viscosities in a centrifugal pump was studied. The results showed that the decrease in turbo performance is mainly caused by the increase in wall shear stress. In Li (6), the conveying performance and internal flow losses of the pump when conveying media of different viscosities were studied. The results showed that the increase in the friction losses between the front and rear cover plates and the outer disk of the impeller and the increase in hydraulic loss in the pump runner is caused by high viscosity. In Yuan et al. (7), the pressure pulsation of the impeller and volute in the runner under design conditions was studied. The results showed that with the increase of radius, the peak of the pressure pulsation in the impeller increases and is maximized at the outlet. However, the numerical value in the diffuser decreases gradually. In Jia et al. (8), a dynamic pressure test was conducted on the centrifugal pump at a given viscosity. The results showed that the pressure of the volute close to the tongue first increases rapidly and then slowly at a low flow rate; however, the pressure decreases sharply at a high flow rate. In Sinha et al. (9), the occurrence and development of the rotating stall of a centrifugal pump with a vane diffuser were studied by particle image velocimetry (PIV) and pressure fluctuation measurement. The results showed that, under design conditions, there is a consistently high-speed leakage flow in the clearance between the impeller and the diffuser from the outlet side to the beginning of the volute. The separation of the leakage flow from the diffuser vane causes the start of the stall. The leakage rate and the velocity distribution in the clearance depend on the direction of the impeller vane. The results (10) showed that the unsteady flow force exerted by the viscous force of the fluid on the impeller is related to the number of vanes and shows a star distribution. Zhang et al. (11) found that, under rotating stall conditions, there are multiple viscous vortex structures in the impeller of the pump. These viscous vortex structures develop with the development of the rotating impeller. Some vane channels are seriously blocked, showing strong unsteady characteristics. In terms of the study of the internal flow characteristics of the food conveying pump, in Zhang et al. (12), the distribution of the boundary vorticity flux (BVF) at the suction surface and the pressure surface of the two pumps was analyzed. The results showed that the optimization design based on BVF diagnosis helps inhibit the bad flow of the centrifugal pump and improve its hydraulic performance. The results (13) showed that the BVF-based flow field diagnosis helps effectively capture the unsteady flow in the pump and optimize the design of the pump impeller by adjusting geometric parameters and modifying the vane shape. In Gu et al. (14), the influence of the static and dynamic interferences of the main vane and splitter vane in the pump and the volute tongue on the internal flow characteristics of the pump was studied; and the energy loss and vorticity distribution in the middle section of the pump were analyzed. The results showed that, after vane cutting, the energy loss and vorticity around the tongue increase obviously; and the splitter vane produces more unsteady energy dissipation than the main vane. In Ji et al. (15), the influence of the distance between the impeller and the guide vane on the internal flow loss distribution and total power loss was studied by entropy generation based on numerical results. In Cui and Zhang (16) and Li et al. (17), entropy generation was used for the numerical analysis of the energy loss of the centrifugal pump; besides, the energy distribution of pressure pulsation and vibration at different flow rates was evaluated according to the experimental results. In Xu et al. (18), the effects of low-frequency ultrasound (US) induced conformational variation of duck liver globular proteins (DLGPs) on the binding behavior of n-alkanes were investigated. This work suggests the great potential of specific conformational variations of DLGPs induced by ultrasonic pretreatments to modulate flavor features of protein-based products. In Sun et al. (19), the present review summarizes the recent advances in the HC (hydrodynamic cavitation)-based pretreatment of LCB (lignocellulosic biomass). The principle of HC is introduced, and the enhancement mechanism of HC is analyzed. In Xuan et al. (20), various synthetic methods for preparing low-dimensional metal-organic frameworks (LD MOFs) are summarized. The synthesis principle and catalytic performance of LD MOFs were explored. For the first time, genetic algorithms (GA) and computational fluid dynamics (CFD) are combined to study and determine the optimal structure of the representative ARHCR (advanced rotational hydrodynamic cavitation reactors) cavity-generating unit (21). In conclusion, most of the previous studies focused on conventional and single fluid media. Relatively few studies compared the influence of fluid food of different viscosities on the internal flow characteristics and conveying performance of the conveying pump. Therefore, it is necessary to study the influence of fluid food of different viscosities on the internal flow characteristics and conveying performance of the conveying pump.

## Centrifugal Pump Model

**FIGURE 1:**
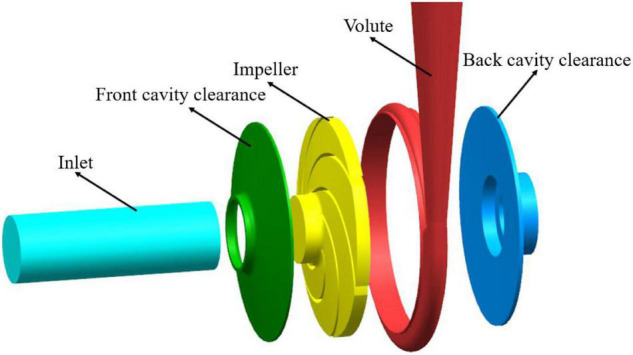
Diagram of the computational domain of flow field.

In this study, the speed of the model pump, *n* = 2,950 r/min; the design flow, *Q*_*d*_ = 11 m^3^/h; and the rated lift, *H*_*d*_ = 38 m. The pump inlet diameter, *D*_*s*_ = 50 mm; the outlet diameter, *D*_*o*_ = 40 mm; the impeller inlet diameter, *D*_1_ = 50 mm; the impeller outlet diameter, *D*_2_ = 160 mm; the impeller outlet width, *b*_2_ = 10 mm; the number of impeller blades, Z = 6; the blade inlet angle, β_1_ = 23°; the blade outlet angle, β_2_ = 23°; the blade thickness, δ = 2.7 mm; the volute base circle diameter, *D*_*b*_ = 165 mm; and the volute inlet width, *b*_2_ = 15 mm. The model pump is shown in Figure 1. The main geometric parameters of the model pump are listed in Table 1.

**TABLE 1:** Performance parameters and geometric parameters of the centrifugal pump.

Parameter/Symbol	Value	Parameter/Symbol	Value
Head/*H*_des_	40 m	Rotate speed /*n*_des_	2,950 rpm
Flow rate/*Q*_des_	10.8 m^3^/h	Specific speed /*n*_s_	37.08
Pump outlet diameter/*D*_o_	40 mm	Impeller inlet diameter /*D*_1_	50 mm
Blade thickness/δ	2.7 mm	Impeller outlet diameter /*D*_2_	160 mm
Blade inlet angle/β_1_	23°	Volute inlet diameter /*D*_b_	165 mm
Blade outlet angle /β_2_	23°	Blade outlet width / *b*_1_	10 mm
Blade number /*Z*	6	Volute inlet width /*b*_2_	15 mm
			

In this study, the same centrifugal pump was used to convey water, glycerin, 67.2 °Bx wild jujube juice, and 71.0 °Bx haw juice of four different viscosities by numerical simulation. The density and viscosity of such food at 25°C are listed in Table 2.

**TABLE 2:** Density and viscosity of four kinds of food liquid at 25°C.

Food liquid	Density/(kg/m^3^)	Dynamic viscosity/(mPa⋅s)
Water	1000.0	0.8949
Glycerin	1261.7	56.0
Wild jujube juice	1339.6	167.0
Hawthorn juice	1350.4	260.4

## Numerical Calculation Method and Test Apparatus

### Numerical Calculation Method

The turbulence model in this study was SST *k–ω* model. When the compressibility of the fluid is not considered, this model can be expressed as follows (22, 23):


$$\frac{\partial \left(\rho \,k\right)}{\partial t}+\frac{\partial \left(\rho \,k\,u_{i}\right)}{\partial x_{i}}=\frac{\partial }{\partial x_{j}}\,\left[\left(\mu +\frac{\mu_{t}}{\sigma_{k}}\right)\,\frac{\partial k}{\partial x_{j}}\right]$$

(1) $$+G_{k}+\rho \,k\,\omega \,\beta^{*}$$

$$\frac{\partial \left(\rho \,\omega \right)}{\partial t}+\frac{\partial \left(\rho \,\omega \,{\bar{u}}_{i}\right)}{\partial x_{i}}=\frac{\partial }{\partial x_{j}}\,\left[\left(\mu +\frac{\mu_{t}}{\sigma_{\omega }}\right)\,\frac{\partial \omega }{\partial x_{j}}\right]+\frac{\partial_{\omega }}{k}\,G_{k}$$

(2) $$-\rho \,\omega^{2}\,\beta +2\,\left(1-F_{1}\right)\,\rho \,\frac{1}{\omega \,\sigma_{\omega }}\,\frac{\partial k}{\partial x_{j}}\,\frac{\partial \omega }{\partial x_{j}}$$

(3) $$\mu_{t}=\frac{\rho \,k}{\omega }$$

where σ_*k*_ = 0.5, σ_*w*_ = 0.5, β = 0.075, β* = 0.09, α = 5/9.

Boundary vorticity flow (BVF) is the core concept of boundary vorticity dynamics. It reflects the rate of boundary vorticity generation and refers to the vortex flux entering the fluid through a unit area in unit time. This concept was proposed by Lighthill in 1963 (24) and was defined as follows:


(4) $$\sigma =v\,\frac{\partial \omega }{\partial n}$$

where **σ** is the value of BVF, **ω** is the vorticity, ν is the kinematic viscosity coefficient, and ***n*** is the unit vector in the normal direction outside the fluid surface.

For a general centrifugal pump, when the vane rotates uniformly, the fluid cannot be compressed, the Reynolds number is great, there is no slip on the wall, and the body force is ignored (25). BVF only has the component produced by the pressure gradient **σ** *_*p*_*,


(5) $$\sigma =\sigma_{p}=\frac{1}{\rho }\,n\times \nabla p$$

Entropy generation (EGR) is a dissipative effect of energy loss, which cannot be avoided in the process of energy conversion. According to the thermodynamic definition, the irreversible process is inevitably accompanied by entropy generation. For the interior of the centrifugal pump, when the heat transfer is not considered, the viscous force in the boundary layer near the wall will cause the mechanical energy in the fluid to be converted into internal energy, which is irreversible; the turbulent pulsation in the high Reynolds number region will cause energy loss and entropy generation. Therefore, the entropy generation theory was used in this study for the study and analysis of the energy loss in the centrifugal pump flow.

For turbulent flow, entropy generation is divided into two parts, namely, entropy generation caused by time-averaged movement and entropy generation caused by velocity fluctuation (23, 26).


(6) SD′′′•′′′=SD¯′′′•′′′+SD′′′′•′′′

where SD¯′′′•′′′ is the entropy generation caused by time-averaged movement, and SD′′′′•′′′ is the entropy generation caused by velocity fluctuation.

The entropy generation caused by time-averaged movement can be calculated using the following formula:


SD¯′′′•′′′=μT{2[(∂⁡u¯∂⁡x)2+(∂⁡v¯∂⁡y)2+(∂⁡w¯∂⁡z)2]+

(7) $$\left[{\left(\frac{\partial \bar{v}}{\partial x}+\frac{\partial \bar{u}}{\partial y}\right)}^{2}+{\left(\frac{\partial \bar{w}}{\partial x}+\frac{\partial \bar{u}}{\partial z}\right)}^{2}+{\left(\frac{\partial \bar{v}}{\partial z}+\frac{\partial \bar{w}}{\partial y}\right)}^{2}\right]\}$$

The entropy generation caused by velocity fluctuation can be calculated using the following formula:


SD′′′′•′′′=μT{2[(∂⁡u′∂⁡x)2+(∂⁡v′∂⁡y)2+(∂⁡w′∂⁡z)2]+

(8) $$\left[{\left(\frac{\partial v^{\prime }}{\partial x}+\frac{\partial u^{\prime }}{\partial y}\right)}^{2}+{\left(\frac{\partial w^{\prime }}{\partial x}+\frac{\partial u^{\prime }}{\partial z}\right)}^{2}+{\left(\frac{\partial v^{\prime }}{\partial z}+\frac{\partial w^{\prime }}{\partial y}\right)}^{2}\right]\}$$

Since the entropy generation caused by velocity fluctuation cannot be directly calculated in the numerical calculation model selected in this study, Kock (27) proposed calculating it with the following formula:


(9) SD′′′′•′′′=ρ⁢εT

where ρ is the medium density, and ε is the turbulent dissipation rate.

Therefore, the total entropy generation in the flow field can be obtained by performing volume integral on it.


(10) SD¯•D¯=∫VSD¯′′′•′′′d⁢V

(11) SD′•D′=∫VSD′′′′•′′′d⁢V

**FIGURE 2:**
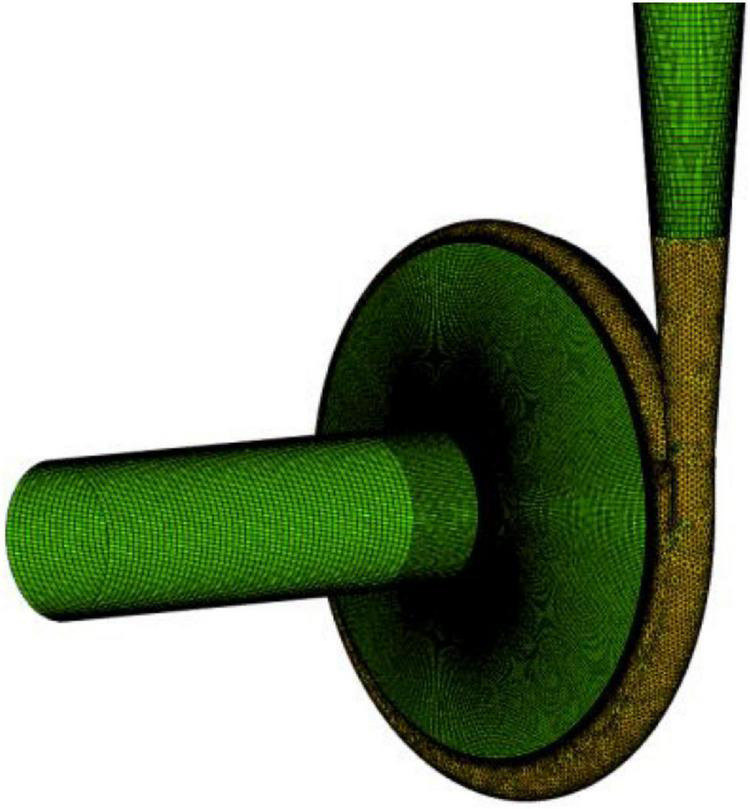
Diagram of flow field domain.

**FIGURE 3:**
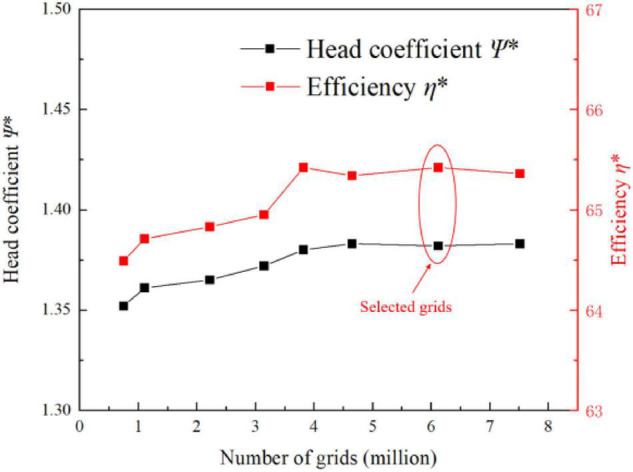
Diagram of flow field mesh independence.

In this study, the whole flow field of the centrifugal pump was numerically calculated by the inlet velocity and the free outlet velocity. The walls in the impeller flow area and the wall in contact with the impeller solid area were set as rotating walls. The walls in other flow domains were set to fixed walls. All walls were set to be smooth. The coupling between speed and pressure was realized by the SIMPLEC algorithm. Second-order upwind and central difference schemes were adopted for the spatial dispersion of convective and diffusive terms, respectively. In the unsteady calculation of the whole flow field, the time step of every 1° rotation of the impeller was taken as a time step, and a rotation cycle contained 120 time steps in total. The speed of the model pump was 2,950 r/min; the time step was 1.69492 × 10^–4^ s; and the convergence residual accuracy of each physical quantity was set to 10^–5^. Figure 2 shows the diagram of the flow field domain and mesh. Figure 3 shows the mesh independence verification diagram of the flow field domain. According to mesh independence verification, the number of mesh cells finally selected herein for the flow field was 6.12 × 10^6^. The head coefficient ψ* is 1.382, and the efficiency coefficient η* is 65.42.

### Test Apparatus

**FIGURE 4:**
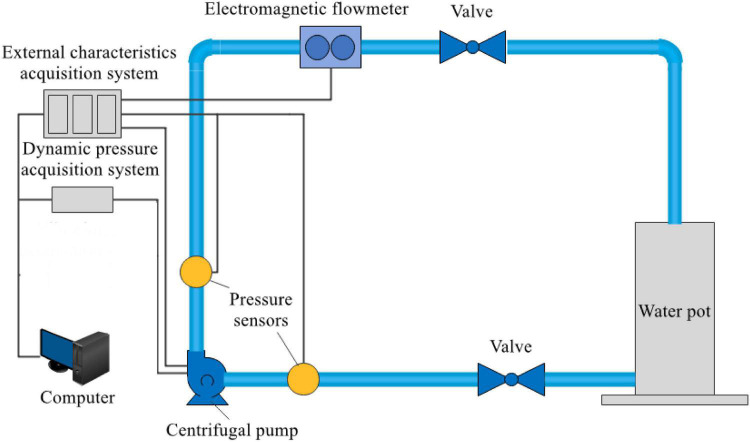
Testing system of the centrifugal pump.

**FIGURE 5:**
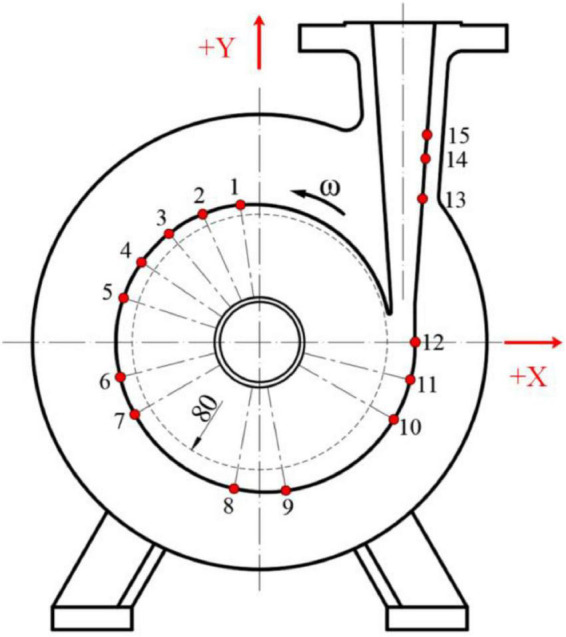
Installation position of the pressure sensor.

The test bench for the operation stability of the centrifugal pump designed in this study is mainly composed of a characteristic data acquisition systems, a centrifugal pump, water tank, high-precision electromagnetic flowmeter, valve, inlet and outlet water pipes, and motor. Figure 4 shows the circuit of the test system for the centrifugal pump. The test object in this study was a low-specific speed centrifugal pump at 2,950 r/min. The installation position of the GYS-I dynamic pressure sensor is shown in Figure 5 and Table 3. The measurement accuracy of the GYS-I pressure sensor is 0.2, the range is –0.1∼1.0 MPa, and the response frequency is 10,000 Hz. (On the premise of ensuring that effective data can be obtained, monitoring points should be arranged in key locations with large pressure changes as far as possible to ensure accurate judgment of the performance of the food delivery pump. This explains why the pressure test points are asymmetrical on the casing).

**TABLE 3:** Installation position of the pressure sensor.

No.	Location	No.	Location
1	θ = 8°	9	θ = 190°
2	θ = 24°	10	θ = 240°
3	θ = 40°	11	θ = 255°
4	θ = 56°	12	θ = 270°
5	θ = 72°	13	h = 90 mm
6	θ = 104°	14	h = 115 mm
7	θ = 120°	15	h = 130 mm
8	θ = 170°		

*Angle θ represents the included angle between the line between the center of the measurement point and the origin and the positive direction of the Y-axis. H represents the vertical height between the centerline of the pressure measuring hole at the outlet and the X-axis of the pump.*

## Results and Discussion

**FIGURE 6:**
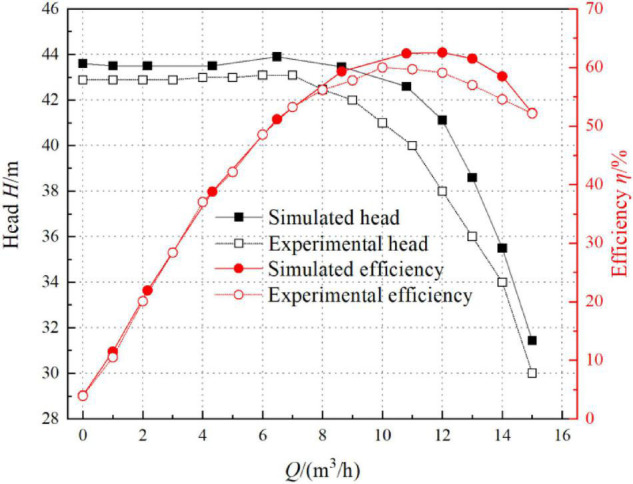
Comparison of external characteristics between simulated and experimental values.

Figure 6 shows the comparison diagram for the simulated value and experimental value of the food conveying pump. The maximum error of the head and efficiency were 7.6 and 7.3%, respectively. According to Figure 6, the simulated value was slightly higher than the actual experimental value. The reason for this is that there may be mechanical seal leakage in practice. Such leakage will cause a decrease in the volumetric efficiency of pumps. Moreover, the wall of the flow parts is rough rather than completely smooth during machining. Besides, energy loss may be caused by the friction between flowing fluids and solid parts. Therefore, both the head and efficiency were slightly lower than the simulated value. Generally speaking, the results of the numerical simulation calculation were consistent with the experimental results, proving the accuracy of the numerical simulation calculation method adopted herein.

**FIGURE 7:**
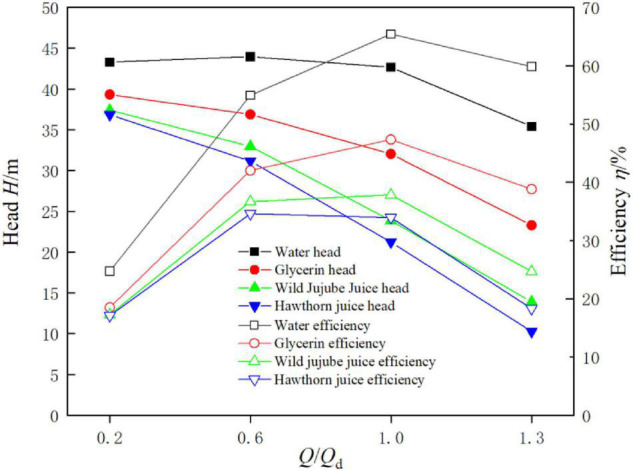
External characteristics of the centrifugal pump with different viscosity.

Figure 7 shows the external characteristic curve of the conveying pump for fluid food of four different viscosities at the flow rate of 0.2 *Q*_*d*_, 0.6 *Q*_*d*_, 1.0 *Q*_*d*_, and 1.3*Q*_*d*_. It can be seen from Figure 7 that the head and efficiency of the pump were the highest when conveying water and the lowest when conveying haw juice. This indicates that the lower the viscosity of fluid food, the closer the conveying performance of the pump to the performance when conveying an eater; the greater the viscosity, the greater the difference. The head and efficiency of the pump gradually decreased with the increase in fluid food viscosity. Under design conditions, the head was 42.66 m, 32 m, 23.88 m, and 21.24 m, respectively, and the efficiencies were 65.42, 47.32, 37.83, and 34.59%, respectively. It can be seen that the higher the viscosity of the medium, the lower the head and the lower the hydraulic efficiency, which is mainly caused by the friction loss of the high viscosity fluid.

**FIGURE 8:**
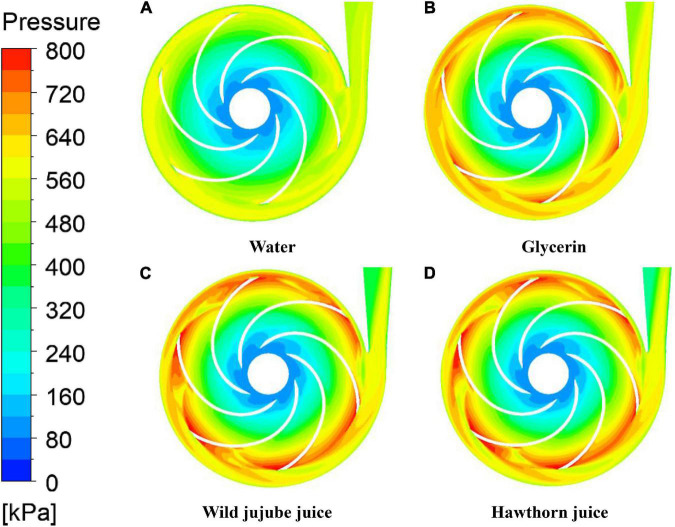
Total pressure of the centrifugal pump with different viscosity. **(A)** Water, **(B)** Glycerin, **(C)** Wild jujube juice and **(D)** Hawthorn juice.

Figure 8 shows the total pressure distribution of fluid food of different viscosities on the midsection of the centrifugal pump under design conditions. According to Figure 8, the pressure distribution in the pump is relatively uniform when conveying water. However, the pressure distribution in the pump is not uniform when conveying glycerin, wild jujube juice, and haw juice. With the increase in viscosity, the high-pressure area near the volute outlet gradually shifted toward the side away from the tongue, resulting in an increase in the pressure gradient on that side. Flow separation occurred at the tongue after the fluid impacted the tongue. Consequently, the pressure near the wall of the volute was lower, and the area of the low-pressure area also increased gradually with the increase in viscosity. The friction resistance of the medium also increased with the increase in fluid viscosity. In addition, at the same volume flow rate, the mass flow rate increased with the increase in fluid density, and the centrifugal force generated by the mass fluid at the same speed was also great, resulting in greater pressure. The friction resistance generated by the viscosity will offset the centrifugal force generated by the fluid during the rotation of the impeller. It can be seen from the figure that, at the design flow rate, the impact of density on pressure is greater than that of viscosity on pressure. This explains why the greater the viscosity, the greater the total pressure.

**FIGURE 9:**
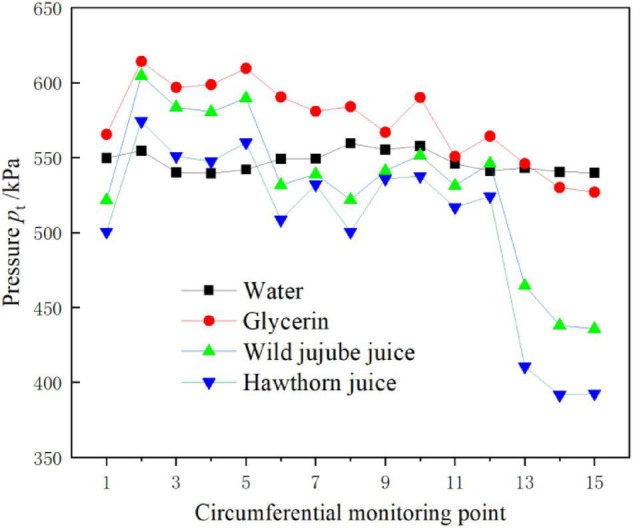
Total pressure on volute casing of the centrifugal pump with different viscosity.

Figure 9 shows the circumferential distribution of total pressure on the wall of the volute at the design flow rate when conveying four food fluids of different viscosities. As shown in the figure, the circumferential pressure on the volute wall changed irregularly with the increase in the density and viscosity of the medium. The circumferential pressure on the volute wall conveying water showed no great fluctuations. However, when conveying glycerin, wild jujube juice, and haw juice, the pressure at the same monitoring point decreased with the increase in viscosity. This is consistent with the pressure distribution on the volute wall as shown in Figure 8. The pressure decreased with the increase in viscosity at monitoring points at the pump outlet away from the impeller and diaphragm (monitoring points 13–15). Nevertheless, the pressure decreased with the increase in viscosity. The higher the viscosity, the greater the decrease in pressure.

**FIGURE 10:**
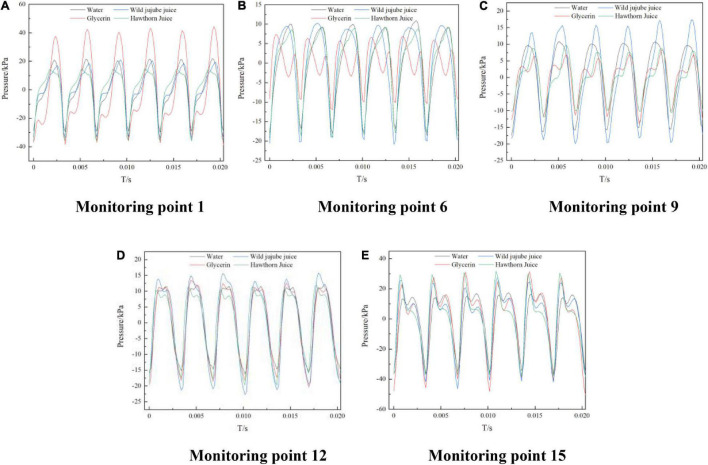
Time-domain distribution of pressure pulsation on volute casing of the centrifugal pump with different viscosities. **(A)** Monitoring Point 1, **(B)** Monitoring Point 6, **(C)** Monitoring Point 9, **(D)** Monitoring Point 12 and **(E)** Monitoring Point 15.

Figure 10 shows the time-domain diagram of pressure pulsation over time at five representative monitoring points on the circumferential wall of the volute. At different viscosities, the pressure pulsation at each monitoring point showed a certain periodicity, there were 6 obvious peaks and troughs, and the number was exactly the number of blades of the impeller. The difference was the difference in pressure amplitude corresponding to the peaks and troughs. This further indicates that the pressure pulsation was significantly affected by the interference of the blade tongue. Monitoring point 1 was the first monitoring point along the helix from the separator. After the impact of the fluid flow and the separator, a great flow separation occurred; and the influence of the separator on the pressure pulsation was shifted to monitoring point 1. The pulsation amplitude of glycerin at this point was the greatest, reaching about 40 kPa; at monitoring points 6 and 9, far away from the tongue, the pressure pulsation amplitude of haw juice was the greatest, and the amplitude of glycerin was the smallest; the pressure pulsation curves of the four fluids at monitoring points 12 and 15 were closer, indicating that these points were mainly affected by the dynamic and static interferences of the tongue, rather than the viscosity.

**FIGURE 11:**
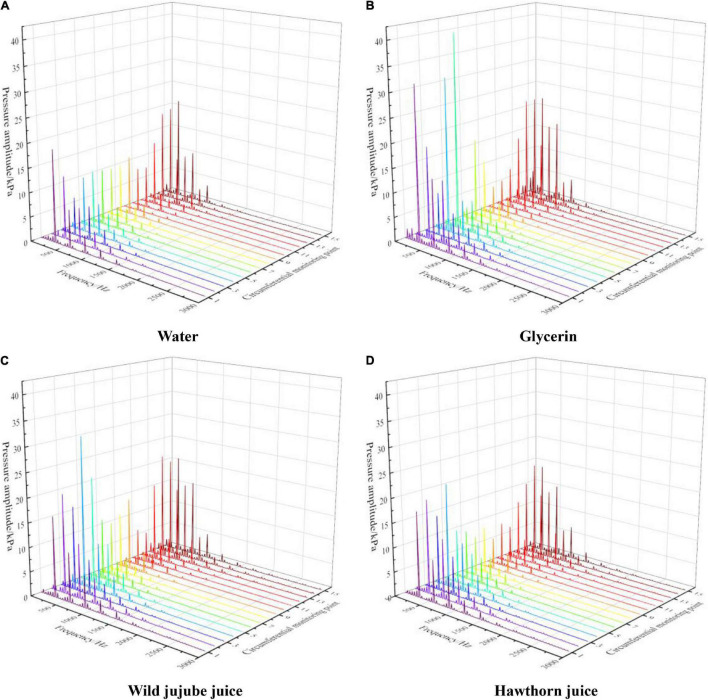
Frequency domain distribution of pressure pulsation on volute casing of the centrifugal pump with different viscosities. **(A)** Water, **(B)** Glycerin, **(C)** Wild jujube juice and **(D)** Hawthorn juice.

To express the pressure pulsation rules at each monitoring point more clearly, a fast Fourier transform (FFT) was performed on the pressure pulsation value at each point to analyze the frequency domain characteristics of pressure pulsation at each monitoring point in the centrifugal pump. Figure 11 shows the frequency domain diagram for the circumferential pressure pulsation at all monitoring points on the volute wall. It can be seen that the pressure pulsation amplitude at each monitoring point appeared at the blade frequency and its frequency was doubled. The amplitude at the blade frequency of *f* = 295 Hz was the greatest. For the same fluid, the pressure pulsation amplitude at the blade frequency of the monitoring point near the circumferential wall of the volute was greater than that of the monitoring point far from the tongue of the volute. The pulsation amplitude at each monitoring point showed a downward trend on the whole with the increase in viscosity, which also indicates that the flow near the circumferential wall of the volute became more stable with the increase in viscosity.

**FIGURE 12:**
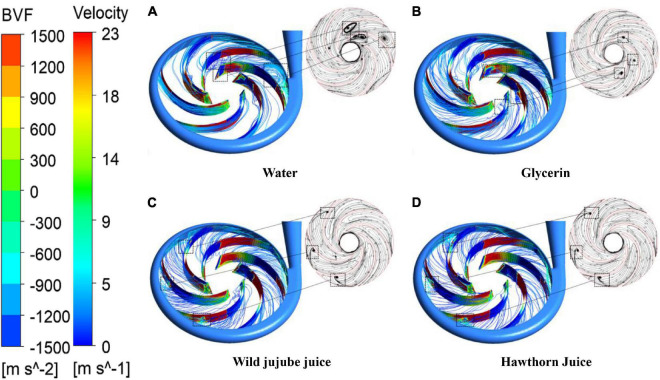
BVF distribution on blades and streamline distribution in the impeller channel with different viscosities. **(A)** Water, **(B)** Glycerin, **(C)** Wild jujube juice and **(D)** Hawthorn juice.

In viscous fluid mechanics, a vortex is one of the main causes of the energy loss of fluid flow. Therefore, the study of the viscous flow (especially secondary flow) in the centrifugal pump cannot be separated from the movement of vortices. It is generally recognized that the peak of BVF causes the peak of downstream boundary vorticity. Therefore, the pressure surface and suction surface of blades were analyzed by BVF in this study. Figure 12 shows the BVF distribution diagram and velocity streamline for the blade of four fluids. According to the figure, the maximum of BVF appeared near the blade inlet where the blade contacted the fluid for the first time and the flow was unstable. The peak of BVF might be caused by the unreasonable design of the blade inlet placement angle. It can be seen from Figure 12 that large or small vortexes appeared in the streamlines flowing through the area with alternating BVF positive and negative peaks, indicating that there was a large flow separation. The vortexes in the impeller flow domain with water flow were derived from the flow separation of the blade inlet near the suction surface and the pressure surface near the tongue. The vortices of the other three fluids were mainly generated on the suction surface of the blade, and the vortex structure was gradually transferred to the impeller outlet with the increase in viscosity.

**FIGURE 13:**
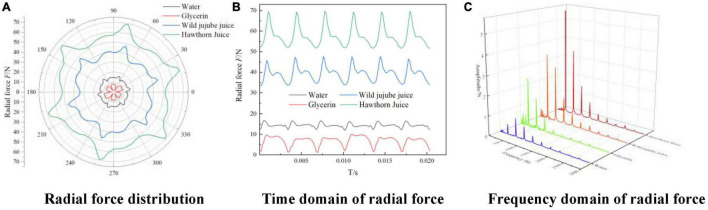
Time-frequency domain distribution of impeller radial force with different viscosities. **(A)** Radial force distribution, **(B)** Time domain of radial force and **(C)** Frequency domain of radial force.

Figure 13A shows the radial flow force vector distribution diagram received by the impeller during a rotating period at the design flow rate in the rotating coordinate system for four different types of fluid food with different viscosities. Figure 13B shows the time-domain diagram of the radial flow force received by the impeller for media with different viscosities at the design flow rate. It can be seen from Figures 13A,B that the radial force received by the impeller for four fluids showed very regular periodic fluctuation, the number of peaks and troughs was consistent with the number of the centrifugal pump blades, and the transient radial force trajectory of the centrifugal pump impeller is symmetric about the central axis as a whole.

With the increase in medium density and viscosity, the radial force of the impeller showed irregular changes; the radial force for glycerin was smaller than that for water, while the radial force for wild jujube juice and haw juice increased with the increase in medium density and viscosity. The density of wild jujube juice and haw juice was close; the radial force was mainly affected by the viscosity, which caused different inlet and outlet pressures, thus causing different radial forces. Since the radial force came from the reaction force of the diffuser to the impeller through fluids, according to 12,The vortex structures in the impeller passage for water were more distributed and asymmetrical under design conditions. Such asymmetric flow caused an uneven force on the runner, resulting in a great radial force. For fluids with a high viscosity, the radial force showed obvious unilateral irregular changes with the medium viscosity. For fluids with low viscosity, however, the radial force showed irregular changes with the medium viscosity.

Figure 13C shows the frequency domain diagram for the radial force on the impeller for media of different viscosities at the design flow rate. Since the speed of the conveying pump was 2,950 r/min, the theoretical shaft frequency was 2,950/60 = 49.17 Hz. Meanwhile, the pump had 6 blades. Therefore, its theoretical blade passing frequency (blade frequency) was 295 Hz. According to Figure 13, the main component of the frequency was blade frequency of *f* = 295 Hz and its frequency doubling. At blade frequency of *f* = 295 Hz, the amplitudes were 0.5133 N, 2.5226 N, 3.4654 N, and 5.5537 N, respectively. The amplitude at the blade frequency also increased with the increase in viscosity. It can be seen from Figures 13A,B that the radial force for glycerin was relatively small, but it showed great fluctuations. As a result, the amplitude at the blade frequency was greater than that for water.

**FIGURE 14:**
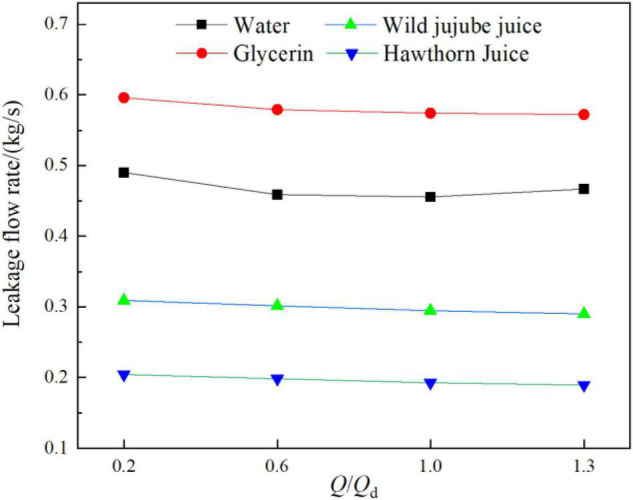
Leakage of the front cavity of a centrifugal pump with different viscosities.

Figure 14 shows the diagram of the leakage rate in the ante-chamber of the centrifugal pump for fluid food of different viscosities under design conditions. Generally, the leakage rate decreased gradually due to the positive proportion of the leakage rate in the clearance to the pressure difference between both ends. When the flow rate increased, the pressure at the impeller outlet gradually decreased, reducing the pressure difference between both ends of the ante-chamber flow domain. This resulted in a decrease in the leakage rate. Under the same conditions, the higher the viscosity, the lower the leakage rate. According to Figure 12, under design conditions, there were more vortex structures in the impeller passage for water, reducing the pressure difference between both ends of the ante-chamber flow domain and resulting in a lower leakage rate for water than that for glycerin. This is consistent with the law of radial force and further indicates that the leakage rate has a certain influence on the radial force. The leakage rate in the ante-wear-ring clearance for water, glycerin, wild jujube juice, and haw juice at the design flow was 0.4556 kg/s, 0.5741 kg/s, 0.2946 kg/s, and 0.1928 kg/s, respectively.

**FIGURE 15:**
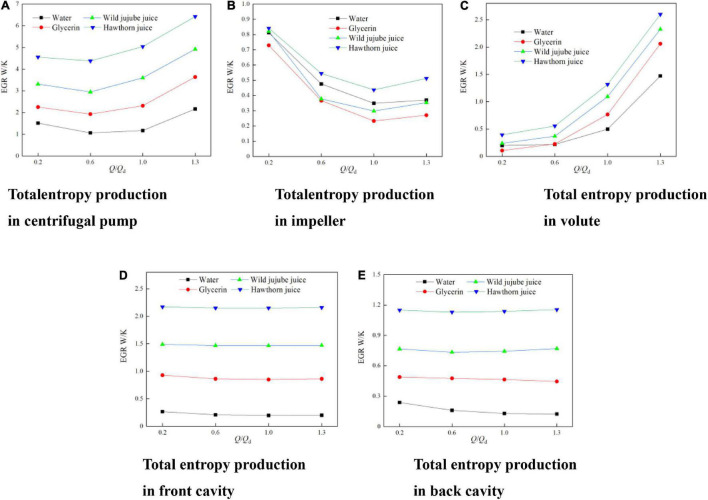
Entropy production in each fluid domain of centrifugal pump with different viscosities. **(A)** Total entropy production in centrifugal pump, **(B)** Total entropy production in impeller, **(C)** Total entropy production in volute, **(D)** Total entropy production in front cavity and **(E)** Total entropy production in back cavity.

To analyze and compare the influence of fluid food of different viscosities on the flow losses in each flow domain of the conveying pump, the whole flow field in the pump was divided into four parts, namely, the impeller, the volute, ante-chamber, and rear-chamber flow domains. Figure 15 shows the comparison diagram for the total entropy generation in each flow domain of the pump at different viscosities of fluid food. According to Figures 15B,C, the entropy generation in the centrifugal pump is mainly concentrated in the impeller flow domain at low flow rates, indicating that the complicated unsteady flow in the impeller is the main factor causing entropy generation; the entropy generation in each flow domain decreases with the increase of the flow rate, making the flow in each flow domain more orderly; and the total entropy generation is minimized near the design flow rate. Then, as the flow rate increases from 1.0*Q*_*d*_ to 1.3*Q*_*d*_, the entropy generation in the volute increases sharply. At this time, the flow loss is mainly concentrated in the volute. In combination with Figure 12, since there is a large vortex structure in the impeller passage of water and the energy loss caused by the vortex structure is much greater than the dissipation caused by viscosity. As a result, the entropy generation in the impeller flow domain of water is greater than that of glycerin and wild jujube juice. According to Figures 15D,E, the entropy generation in the pump chamber is less affected by flow rate and more by fluid food viscosity. With the increase in viscosity, the proportion of the total entropy generation in the pump chamber increases. The proportion of the entropy generation in the pump chamber of fluid food of four different viscosities at the design flow rate is 27.84, 56.80, 61.37, and 65.21%, respectively. This indicates that the flow loss in the pump chamber is the major source of the flow loss in the centrifugal pump conveying high viscosity fluid.

Under each condition, with the increase in fluid food viscosity, the entropy generation in each flow domain in the pump increases, and the total entropy generation increases accordingly. The total entropy generation of water, glycerin, wild jujube juice, and haw juice at the design flow rate is 1.17 W/K, 2.32 W/K, 3.60 W/K, and 5.04 W/K, respectively. Besides, it indicates that the overall flow loss in the centrifugal pump increases with the increase in fluid viscosity.

**FIGURE 16:**
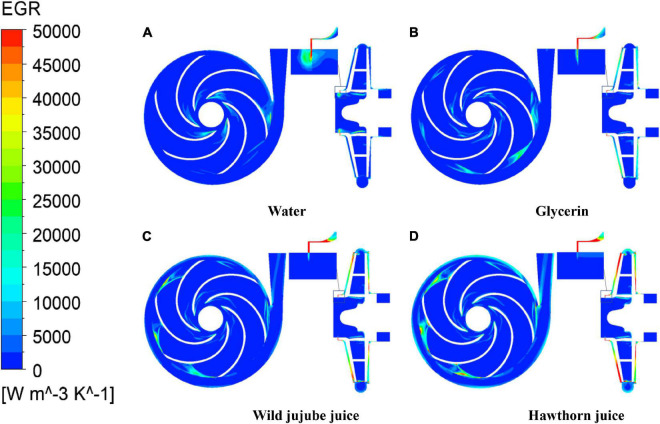
Entropy production distribution of impeller and volute in the middle and *y* = 0 section. **(A)** Water, **(B)** Glycerin, **(C)** Wild jujube juice and **(D)** Hawthorn juice.

Figure 16 shows the entropy generation distribution on the midsection between the impeller and volute of the centrifugal pump and the axial *y* = 0 section under design conditions. On the midsection between the impeller and volute, the high entropy generation areas of water (entropy generation greater than 25,000 W/K/m^3^) are mainly distributed in the impeller inlet and near the tongue; the high entropy generation areas in the impeller of glycerin are mainly distributed in the impeller passage near the vane outlet, especially near the tongue. In the impeller of wild jujube juice, the high entropy generation areas are mainly distributed at the vane outlet of each runner; and the entropy generation begins to appear in a large area near the volute tongue. In the impeller of haw juice, the high entropy generation areas are mainly distributed at the vane outlet of each runner and the volute near the tongue; and with the increase in viscosity, the entropy generation gradually decreases along the spiral line of the volute. This indicates that the fluid impact loss at the impeller inlet and the flow loss caused by dynamic and static interferences between the impeller and volute tongue are major sources of entropy generation. On the axial *y* = 0 section, there are large high entropy generation areas near the wear-ring, the reason for which is that partial fluid at the outlet of the impeller enters the wear-ring clearance through the ante-chamber of the pump and then returns to the pump inlet area. Meanwhile, due to the narrow flow area at the wear-ring clearance, the velocity of the fluid is excited there, which forms a jet when flowing to the inlet, thus aggravating the flow loss. With the increase in viscosity, the flow loss at the wear-ring clearance decreases, while the entropy generation decreases accordingly.

## Conclusion

In this study, the lift, efficiency, and the internal unsteady flow characteristics for four kinds of fluid food of different viscosities (i.e., water, glycerin, 67.2 °Bx wild jujube juice, and 71.0 °Bx haw juice) were investigated. The main conclusions are as follows:

(1) With the increase in viscosity, the flow loss in the conveying pump increases; and the conveying performance for fluid food gets worse. Under design conditions, the head is 42.66 m, 32 m, 23.88 m, and 21.24 m, respectively, and the efficiencies are 65.42, 47.32, 37.83, and 34.59%, respectively. At the design flow rate, the total pressure increases with the increase in viscosity due to the greater influence of density on pressure than that of viscosity.

(2) The circumferential pressure on the volute wall shows irregular changes with the increase in medium density and viscosity. When conveying water, the pressure on the circumferential wall of the volute of the pump shows no great fluctuations. However, with the increase in fluid food viscosity, the pressure in the pump gradually decreases; however, the pressure drop increases.

(3) For fluids with high viscosity, the radial force shows obvious unilateral irregular changes with the medium viscosity. For fluids with low viscosity, however, the radial force shows irregular changes with the medium viscosity. The amplitude of the radial force at the blade frequency also increases with the increase in viscosity. At the blade frequency of *f* = 295 Hz, the amplitudes are 0.5133 N, 2.5226 N, 3.4654 N, and 5.5537 N, respectively.

(4) With the increase in viscosity, the flow loss at the wear-ring clearance decreases, while the entropy generation decreases accordingly. The flow loss in the pump chamber is the major source of the flow loss in the centrifugal pump conveying high viscosity fluid. The proportion of the total entropy production value of the pump cavity increases, and the proportion of the entropy production of the pump cavity under the design flow rate is 27.84%, 56.80%, 61.37%, and 65.21%, respectively.

Overall, the study on food viscosity can provide a useful reference for the efficient and stable operation of food conveying pump.

## Data Availability Statement

The original contributions presented in this study are included in the article/supplementary material, further inquiries can be directed to the corresponding author/s.

## Author Contributions

XJ: methodology, writing – review, and editing. QC: formal analysis, data curation, visualization, and writing – original draft. ZZ: conceptualization. QD: supervision. PG: project administration. All authors contributed to the article and approved the submitted version.

## Conflict of Interest

The authors declare that the research was conducted in the absence of any commercial or financial relationships that could be construed as a potential conflict of interest.

## Publisher’s Note

All claims expressed in this article are solely those of the authors and do not necessarily represent those of their affiliated organizations, or those of the publisher, the editors and the reviewers. Any product that may be evaluated in this article, or claim that may be made by its manufacturer, is not guaranteed or endorsed by the publisher.
